# Yeast‐based reporter assay system for identifying the requirements of intramembrane proteolysis by signal peptide peptidase of *Arabidopsis thaliana*

**DOI:** 10.1002/2211-5463.12936

**Published:** 2020-08-07

**Authors:** Kenta Kusunoki, Masako Hoshi, Tomoko Tamura, Tatsuya Maeda, Keiko Abe, Tomiko Asakura

**Affiliations:** ^1^ Department of Applied Biological Chemistry Graduate School of Agricultural and Life Sciences University of Tokyo Japan; ^2^ Department of Nutritional Science and Food Safety Faculty of Applied Bioscience Tokyo University of Agriculture Tokyo Japan; ^3^ Department of Biology Hamamatsu University School of Medicine Shizuoka Japan

**Keywords:** endoplasmic reticulum, helix breaker, intramembrane cleavage, signal peptide peptidase, yeast‐based reporter assay

## Abstract

Signal peptide peptidase (SPP) is an aspartic protease with two active sites, YD and GXGD, in the transmembrane domain. SPP cleaves signal peptides, and the released fragments play key roles in the immune system, embryo development and protein turnover in cells. Despite SPP having an important function, a general system to identify the requirements of intramembrane proteolysis by SPP has not been developed because proteolysis occurs in the membrane. In this study, we first established a reporter assay system in yeast to verify the cleavage activity of the *Arabidopsis thaliana* SPP (AtSPP). Next, we screened candidate substrates of AtSPP from *A. thaliana* pollen and roots. In the pollen, 13 signal peptides with 'pollen' and 'cell wall' as gene ontology terms were selected. In the roots, mutants overexpressing AtSPP were constructed, and gene expression changes were compared with the wild‐type. Nine signal peptides expressed in the roots were selected. Then we used the candidate substrates in our reporter assay system to determine the requirements for proteolysis by AtSPP. Fifteen of 22 signal peptides were cleaved by AtSPP. The absence of the positively charged amino acids, His and Lys on the C terminus of the signal sequence, was observed in cleaved substrates. Moreover, mutation of a helix breaker‐to‐Leu substitution in the intramembrane region in substrates prevented cleavage by AtSPP. These results indicated that substrates of AtSPP required the helix breaker structure to be cleaved.

## Graphical abstract

We constructed a yeast‐based reporter assay system to verify the cleavage activity of *Arabidopsis thaliana* signal peptide peptidase, a transmembrane protease localized in the endoplasmic reticulum. *Arabidopsis thaliana* signal peptide peptidase cleaved 15 candidate substrates that lacked the positively charged amino acids, His and Lys, in the C‐region of signal peptides. Our assay system may be suitable for identifying innate substrates with crucial roles in plants.

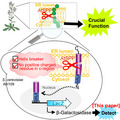

**Abbreviations**
ADE2: adenylosuccinate synthetase
AtSPP: *Arabidopsis thaliana* signal peptide peptidase
ER: endoplasmic reticulum
ERAD: endoplasmic reticulum‐associated degradation
Gal4: galactose‐responsive transcription factor
gpUL40: glycoprotein UL40
HCMV: human cytomegalovirus
HIS3: imidazole‐glycerol phosphate dehydratase
HsSPP: human signal peptide peptidase
MHC: major histocompatibility complex
OsSPP: *Oryza sativa* signal peptide peptidase
SD‐LW: synthetic defined medium lacking Leu and Trp
SPP: signal peptide peptidase

Signal peptide peptidase (SPP), EC3.4.23.B24, which belongs to the intramembrane‐cleaving proteases, is a multitransmembrane aspartic protease, localized in the endoplasmic reticulum (ER), and having YD and GXGD motifs as the active sites in the transmembrane domain [1]. SPP cleaves several type II membrane signal peptides, where the location of the N termini is in the cytosol and the C termini is in the lumen [2]. SPP is expressed in human [1], zebrafish [3], *Drosophila* species [4], malaria [5], yeast [6] and plants, such as *Arabidopsis thaliana* [7] and *Oryza sativa* [8].

Regulated intramembrane proteolysis by SPP plays crucial roles in biological processes [9]. For example, human SPP (HsSPP) cleaves the signal peptide of the major histocompatibility complex (MHC) class I molecule, and the C‐terminal peptide‐epitope is transported to the cell surface as an immunogenic peptide–MHC class I complex, which is inspected by the T cell receptor [1, 10]. Proteolysis by SPP is also linked to protein degradation [11] and canceration [12]. In mice, the SPP substrates unique to mice and the functions of the cleaved fragments have not been discovered because SPP knockout leads to embryonic lethality [13]. Those of *A. thaliana* have also not been discovered because of lethality caused by pollen tube elongation disorder [14]. These findings indicate that SPP plays basic and essential roles in cells. Just as in mice, none of the substrates of *A. thaliana* SPP (AtSPP) have been discovered in plants. Our previous study showed that *AtSPP* is strongly expressed in the shoot meristem and the epidermis of the radicle [7]. SPPs in rice [*Oryza sativa* SPP (*OsSPP*)] are also expressed in the shoot apex [8]. In those tissues, cell division and cell elongation are promoted. The intracellular localization of both AtSPP and OsSPP is in the ER [7, 8]. Our group also showed that SPP possesses proteolytic activity [15]. Although AtSPP is required for pollen tube elongation [14], the functions of the fragments cleaved by SPP in plants are unclear. Furthermore, general systems to verify the requirements of intramembrane proteolysis by SPP have not been developed because of the two‐step regulated proteolysis and because proteolysis occurs in the membrane.

Because some peptide fragments would have a function after cleavage by SPP, construction of a handy assay system to identify the proteolytic activity in SPP could contribute to the following: (a) a better understanding of the enzymatic properties of SPP, including enzymatic activity and requirements for intramembrane proteolysis; (b) identification of the substrates; and (c) understanding the physiological function of processed substrates. In this study, we first constructed a yeast‐based reporter assay system (Fig. 1) to verify the cleavage activity of AtSPP. Next, we screened candidate substrates of AtSPP from the pollen and roots in *A. thaliana*, and then investigated whether the candidate substrates were cleaved by AtSPP, using the constructed reporter assay system. Furthermore, we identified the requirements of intramembrane proteolysis with mutant substrates.

**Fig. 1:**
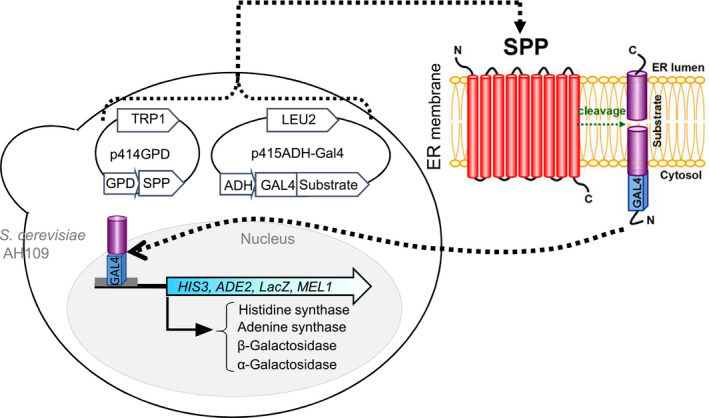
The design of the reporter assay system expressing SPP and its substrate in yeast. Two proteins (SPP and one of the candidate substrates) were expressed from the p414GPD vector and the p415ADH‐Gal4 vector, respectively. In the *Saccharomyces cerevisiae* *Δspp* mutant, *ADE2*, *HIS3*, *LacZ* and *MEL1* reporters were activated only when a candidate substrate was cleaved by SPP; then the N‐terminal Gal4 fused fragment was released into the nucleus and bound to the Gal4‐responsive promoter.

## Materials and Methods

### Construction of expression plasmid vectors

The plasmid vector, p414GPD, was used to express SPPs. The full‐length AtSPP (UniProtKB; O81062) and HsSPP (UniProtKB; Q8TCT9) were cloned into the vector using *Bam*HI and *Eco*RI sites. Insertion of point mutation in the active sites (D198A, D239A) in AtSPP was carried out by inverse PCR using KOD‐plus‐neo (TOYOBO). PCR primers are described in Table S1. The p415ADH vector was used to coexpress AtSPP with the candidate substrates in yeast. *Galactose‐responsive transcription factor* (*Gal4*) was amplified by PCR using pGADT7 AD (Clontech, CA, USA) as a template and ligated into the p415ADH vector using *Spe*I and *Bam*HI sites. The constructed vector was named p415ADH‐Gal4. The signal peptide sequence of human cytomegalovirus glycoprotein UL40 (HCMV gpUL40, P16780) was synthesized by TAKARA Bio Co. Ltd., Shiga, Japan. The single‐strand DNA of the signal peptide sequence of bovine prolactin, preprolactin (UniProtKB; P01239), was synthesized by Eurofins Genomics Co., Ltd. PCR was performed using the earlier fragments as templates. The PCR primers are shown in Table S1. Subsequently, the signal peptide sequences were fused at *Bam*HI and *Hind*III sites in the p415ADH‐Gal4 vector.

### Screening and cloning of genes encoding candidate substrates of AtSPP

#### Screening from pollen

Because the SPP knockout in *A. thaliana* is lethal because of pollen tube elongation disorder [14], genes containing the terms ‘pollen’ and ‘cell wall’ in the gene ontology were selected using The *Arabidopsis* Information Resource (http://www.arabidopsis.org/), and then genes encoding transmembrane proteins and having signal peptides at the 5′‐terminal region were selected using SignalP (http://www.cbs.dtu.dk/services/SignalP/). Details are described in Fig. S1.

#### Screening from the roots

We constructed the AtSPP‐overexpressed lines and performed DNA microarray analysis. Next, genes encoding single‐pass transmembrane proteins were selected using ATTED‐II (http://atted.jp/), and then genes expressed in the roots and having signal peptides were selected. Details are described in Fig. S2.

#### Cloning

Nineteen cDNA clones out of the selected 22 candidate substrates (AT2G06850, AT5G49360, AT1G65590, AT5G07410, AT1G69940, AT3G26720, AT5G25460, AT5G11420, AT4G25900, AT5G21100, AT5G12950, AT4G32460, AT4G33720, AT2G46330, AT4G25810, AT5G61790, AT5G64100, AT5G42020, and AT3G05490) were purchased from the RIKEN BRC through the National Bio‐Resource Project of the MEXT, Japan. The cDNA clone of AT5G54570 was purchased from the *Arabidopsis* Biological Resource (ABRC, Ohio State University). To amplify genes of AT4G12510 and AT1G54000, we extracted RNA from roots of *A. thaliana* using the RNeasy Plant Mini kit (QIAGEN) and synthesized the cDNA using the SuperScript First‐Strand Synthesis System (Thermo Fisher Science) according to each manufacturer’s protocol. The signal peptide sequences of the 22 genes were amplified by PCR using ExTaq (TAKARA Co., Ltd) with the sense and antisense primers as described in Table S2. The PCR products were fused into *Bam*HI and *Hind* III sites in the p415ADH‐Gal4 vector.

### Production of candidate substrates having mutations within the peptides

To insert a point mutation into genes encoding a candidate substrate of AtSPP, we carried out inverse PCR using KOD‐plus‐neo with the sense and antisense primers as described in Table S3. The products were subcloned into p414GPD with the ligation Kit version 2.1 (TAKARA Co., Ltd).

### Cell culture and the yeast‐based reporter assay

The yeast‐based reporter assay system was derived from a previous paper [16]. *Saccharomyces cerevisiae* strain AH109 Δ*spp* (Δ*YKL*100c; P34248) was made by recombination using KanMX4 from strain AH109 [*MATa, trp1‐901, leu2‐3, 112, ura3‐52, imidazole‐glycerol phosphate dehydratase (his3)‐200, gal4Δ, gal80Δ, LYS2::gal1_UAS_‐GAL1_TATA_‐HIS3, GAL2_UAS_‐GAL2_TATA_, ura3::MEL1_TATA_‐lacZ*] to avoid endogenous protease activity. Yeast transformation was performed using Frozen‐EZ Yeast Transformation II (Zymo Research) to introduce the p414GPD vector containing *AtSPP* or *HsSPP*, and the p415ADH‐Gal4 vector containing genes encoding candidate substrates. The products were selected on synthetic defined medium lacking Leu and Trp.

### The yeast spotting assay

The transformed cells were incubated in yeast extract–peptone–dextrose (YPD) medium overnight at 30 °C on a shaker at 300 rotations per minute (rpm), and then the cells were prepared to yield an *A*_600_ of 1 (10^7^ cells·mL^−1^). Subsequently, the cells were diluted to an *A*_600_ of 0.1, 0.01 and 0.001 with sterilized water. Then 5 μL of each culture was spotted on selection synthetic defined medium lacking adenine, His, Leu and Trp containing 20 mg·mL^−1^ X‐α‐gal (TAKARA) and incubated at 30 °C for 3 days. pCL1 [17] was used as a positive control plasmid encoding the full‐length GAL4.

### β‐Galactosidase assay

Colonies on selection synthetic defined medium by the yeast spotting assay were inoculated into 5 mL YPD medium and then incubated at 30 °C on a shaker at 300 rpm. The solution was diluted to yield an *A*_600_ of 0.05 (5 × 10^5^ cells·mL^−1^) with sterilized water. Then 50 μL of cells was added to the mixed solution, which was made of 2 μL Gal‐Screen buffer A and 48 μL Gal‐Screen buffer B in Gal‐Screen System (Applied Biosystems). After incubation of the mixture at 30 °C for 1 h, 75 μL of the mixture was placed in a microplate in a Centro XS3 LB960 (Berthold Technologies), and the luminescence was measured for 0.1 s·well^−1^. The amount of β‐galactosidase corresponding to cleavage enzyme activity was calculated using a standard curve, which was generated with β‐galactosidase (TOYOBO). The results were expressed as the mean ± standard error. Statistical analysis was performed with JMP software (Ver. 12). A statistical difference was determined using a two‐sided Welch’s *t*‐test, and a difference with *P* < 0.05 was considered significant.

## Results

### The detection of AtSPP protease activity by a yeast‐based reporter assay system

Figure 1 shows the design of the yeast‐based reporter assay system in this study. Four genes encoding *HIS3*, adenylosuccinate synthetase (*ADE2*), β‐galactosidase‐expressing *lacZ* and α‐galactosidase‐expressing *MEL1* were controlled by Gal4. When the substrate was cleaved by SPP, the N termini of the fragment that was conjugated to Gal4 were released, moved to the nucleus and then bound to the Gal4‐responsive promoter. Finally, genes controlled by the promoter were expressed.

**Fig. 2:**
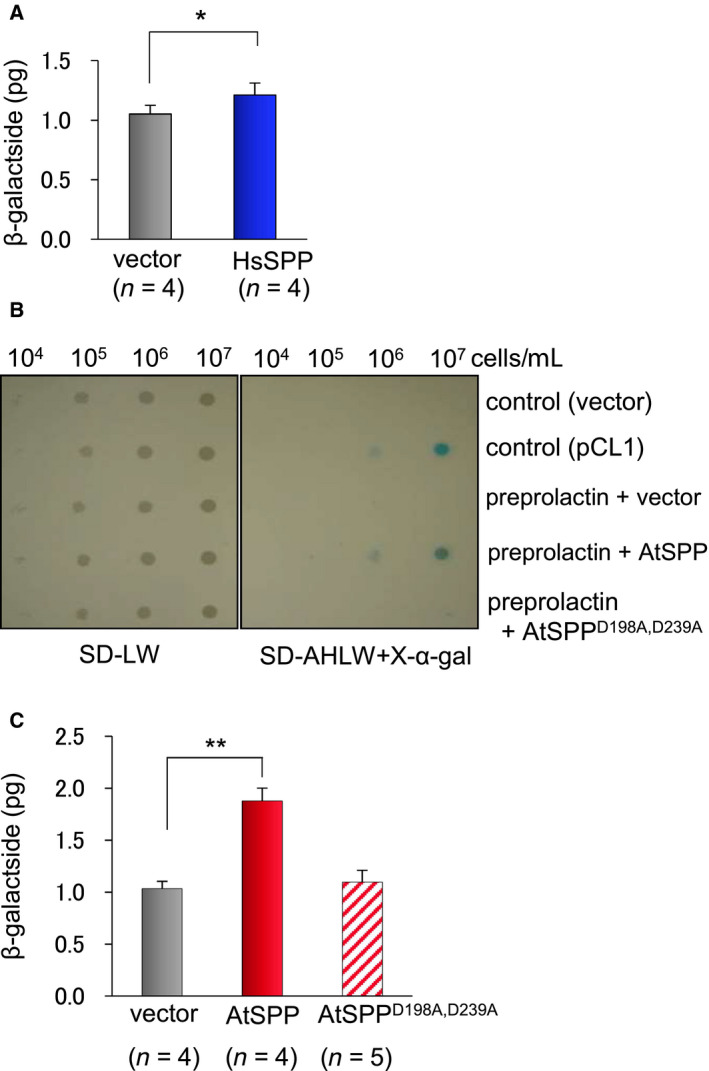
Quantification of signal peptide processing. (A, C) Processing of signal peptides was investigated in the yeast‐based reporter assay and quantified as the amount of β‐galactosidase. Gray bars indicate the amount of β‐galactosidase when the p414GDP vector not containing *AtSPP/HsSPP* was used as a control. The blue bar (A) and the red bar (C) indicate the amount of β‐galactosidase when the p414GDP vector containing *HsSPP* or *AtSPP* was used to cleavage preprolactin. The striped bar indicates the p415ADH‐Gal4 vector containing mutant AtSPP. Results are expressed as the mean ± SEM of four (A) or five (C) trials. The results were analyzed by the two‐sided Welch’s *t*‐test. Asterisks indicate a significant difference of *P < 0.05 and **P < 0.001. (B) Spot assay on the selection medium. The right panel shows colonies on synthetic defined medium containing X‐α‐gal and lacking Ala, His, Leu and Trp. The left panel shows colonies on synthetic defined medium lacking Leu and Trp. pCL1 is a positive control plasmid encoding the full‐length GAL4. Blue colonies are observed when α‐galactosidase expression is induced by GAL4, following secretion of α‐galactosidase from AH109 cells.

Preprolactin is known as a classical substrate of HsSPP. First, we tested the validation of the reporter assay system for the detection of protease activity using recombinant vectors containing *HsSPP* in p414GPD and *preprolactin* in p415ADH. AH109 cells were transformed with those vectors and grown on X‐α‐gal medium lacking Leu and Trp. Compared with the vector not containing *HsSPP*, the amount of β‐galactosidase produced was significantly higher in yeast transformed with the p414GPD vector containing *HsSPP* (Fig. 2A). Because the reporter assay system worked well, we replaced the *HsSPP* gene with the *AtSPP* gene in the p414GPD vector to confirm the protease activity of AtSPP in the assay system. As shown in Fig. 2B, blue colonies were observed when α‐galactosidase expression was induced by Gal4. The amount of β‐galactosidase in cells growing on a synthetic defined medium lacking adenine, His, Leu and Trp containing X‐α‐gal was 1.8 times higher than that in cells transformed with the vector control. AtSPP contains two active sites, D198 and D239. We substituted the two aspartic acids with Ala, and the resulting AtSPP mutant, AtSPP^D198A,D239A^, was tested for processing of preprolactin. The amount of β‐galactosidase produced in cells containing the mutant AtSPP was not different from cells containing the vector control (Fig. 2C).

**Fig. 3:**
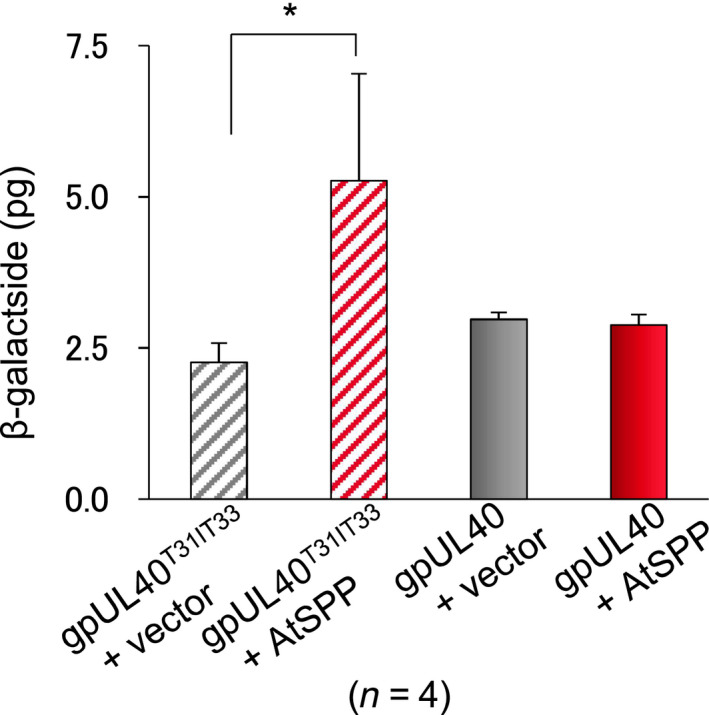
Processing of artificially mutated substrates by AtSPP. Processing of mutated signal peptides was investigated in the yeast‐based reporter assay and was quantified as the amount of β‐galactosidase. Gray bar indicates the amount of β‐galactosidase when the p414GDP vector not containing *AtSPP* was used as a control. Red bar indicates the amount of β‐galactosidase when the p414GDP vector containing *AtSPP* was used to cleavage gpUL40/gpUL40^T31IT33^. The striped bars indicate the p415ADH‐Gal4 vector containing mutant gpUL40. Results are expressed as the mean ± SEM of four trials. The results were analyzed by the two‐sided Welch’s *t*‐test. *P < 0.05.

Signal peptides have a three‐domain structure: an amino‐terminal positively charged region (n‐region, 1–5 residues); a central, hydrophobic part (h‐region, 7–15 residues); and a more polar C‐terminal domain (c‐region, 3–7 residues) [18]. It is known that a disruption of the h‐region either by a charged amino acid or helix breaker leads to a change in the rate of proteolysis [18]. Lemberg [19] described the HCMV gpUL40 that contains two Arg residues (RIR) at the end of the h‐region in the signal peptide (gpUL40^R31IR33^); this signal peptide is not processed by SPP. But they also report that the substitution of those Arg to Thr in the HCMV gpUL40 signal peptide, gpUL (gpUL40^T31IT33^), allowed cleavage by HsSPP. In accordance with that information, we performed the yeast‐based reporter assay using gpUL40^T31IT33^ as an artificial substrate of AtSPP. We observed the significant increase of β‐galactosidase for gpUL40^T31IT33^ as a control and confirmed that the mutant was cleaved by AtSPP (Fig. 3, red striped bar). In accordance with the earlier results, we concluded that the yeast‐based reporter assay system was able to verify the requirements for AtSPP proteolysis.

### Screening candidate substrates of AtSPP

As shown in Table 1, 13 signal peptides associated with pollen function were selected from 212 genes containing the terms ‘pollen’ and ‘cell wall’ in the gene ontology (Fig. S1).

**Table 1:** Signal peptide sequences of candidate substrates of AtSPP. Asterisks indicate signal peptides that were cleaved by AtSPP.

Localization: pollen		
AGI code	Signal peptide sequence	
AT2G06850	NH_2_‐TVSSSPWALMALFLMVSSTMVMAIP‐COOH	*
AT5G49360	NH_2_‐SCYNKALLIGNKVVVILVFLLCLV**H**SSES‐COOH	
AT1G65590	NH_2_‐RGSGAKIAGVL**[P]**LFMLFIAGTISA‐COOH	*
AT5G07410	NH_2_‐RYTNVSILLGMLVIFVSPMVFA‐COOH	*
AT1G69940	NH_2_‐GYTNVSILLGLLMVFVTPMVFA‐COOH	*
AT5G54570	NH_2_‐ESLMRLVLVLFPFFVVFFVPLD**H**VSS‐COOH	
AT3G26720	NH_2_‐AVKCFSLYLILAAIVIGGVTS‐COOH	*
AT5G25460	NH_2_‐EGVTVVSFFLLFIATAMA‐COOH	*
AT5G11420	NH_2_‐KGG**[S]**L**[S]**FLFVLLIATITSVIC‐COOH	*
AT4G25900	NH_2_‐MGNKRNLGFVIFFWALVVAVVA‐COOH	*
AT5G21100	NH_2_‐AVIVWWLLTVVVVAF**H**SASA‐COOH	
AT5G12950	NH_2_‐KSGLIITIALLLYTSSFVLVSVA‐COOH	*
AT4G32460	NH_2_‐KEMGVIVLLLLHSFFYVAFCF‐COOH	
Localization: roots		
AGI code	Signal peptide sequence	
AT4G33720	NH_2_‐KIFNSSQNLFLAITFFLVLIV**H**LKA‐COOH	
AT2G46330	NH_2_‐ASRNSVTGFALFSFVFAVILSLAGA‐COOH	*
AT4G25810	NH_2_‐AMISYSTIVVALLASFMICSVSA‐COOH	*
AT5G61790	NH_2_‐RQRQLFSVFLLLLAFVSFQ**K**LCYC‐COOH	
AT5G64100	NH_2_‐GRGYNLLFVLV**[T]**FLVLVAAVTA‐COOH	*
AT5G42020	NH_2_‐ARSFGAN**[S][T]**VVLAIIFFGCLFAFSTA‐COOH	*
AT3G05490	NH_2_‐TNTRAIYAVIAILAIVISAVES‐COOH	
AT4G12510	NH_2_‐ASKISASLVIFLTFNILFFTLTTA‐COOH	*
AT1G54000	NH_2_‐MANNCNLVSVLCVILVLTLF**H**NPITVAG‐COOH	*

Underbar Indicates positively charged residues located in the c‐region.

Brackets indicate helix breaker amino acid mutated in this experiment.

To select the candidate substrates expressed in the roots, *we* first detected the strong expression of AtSPP in the root (*Arabidopsis* eFP Browser and Tamura et al. [7]). Despite the unique expression, no substrate expressed in the root has been revealed. We then focused on an article [20] that describes the fragment of heme oxygenase 1 cleaved by HsSPP that was transported to the nucleus and enhanced the proliferation and migration of cancer cells. This report led us to consider that mutant lines of AtSPP could change the expression levels of substrates. Therefore, we constructed the AtSPP knockdown and overexpressed lines, and performed DNA microarray analysis. According to the statistical analysis, AtSPP‐overexpressed lines and the Col‐0 were used to compare gene expression changes as the first step of screening. In accordance with the screening flow chart described in Fig. S2, nine signal peptides expressed in the roots were selected (Table 1). Those 22 candidates were subjected to the yeast reporter assay system.

### Cleavage of candidate substrates by AtSPP

**Fig. 4:**
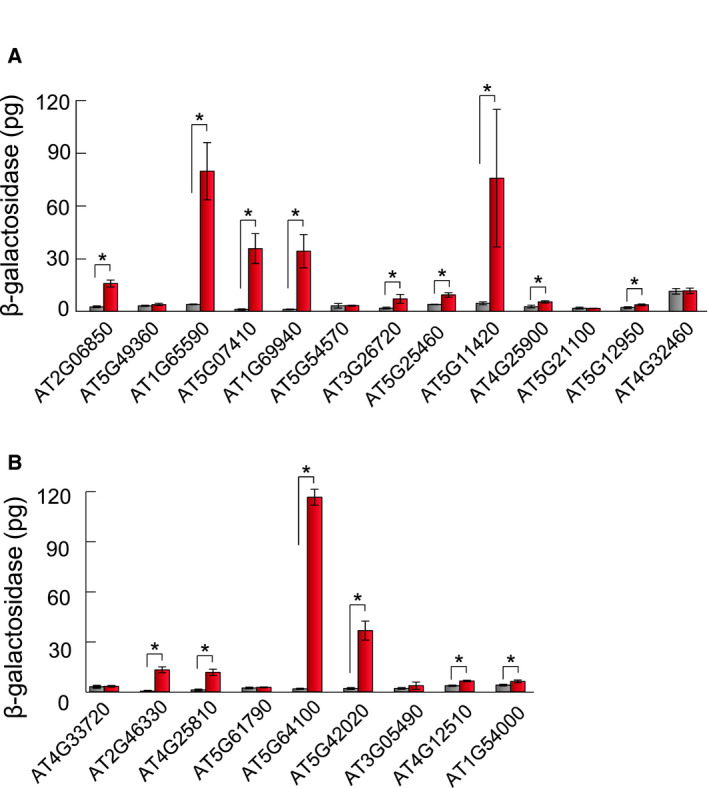
Processing of candidate substrates by AtSPP. Candidate substrates in the pollen (A) and in the roots (B) were expressed with AtSPP in the yeast‐based reporter assay, and cleavage was quantified as the amount of β‐galactosidase produced. Gray bars indicate the amount of β‐galactosidase when the p414GDP vector not containing *AtSPP* was used as a control. Red bars indicate the amount of β‐galactosidase when the candidate substrates were processed by AtSPP from the p414GDP vector containing *AtSPP*. The results are presented as the mean ± SEM of four or five trials. The results were analyzed by the two‐sided Welch’s *t*‐test. Asterisks indicate difference with *P < 0.05.

For candidate substrates expressed in the pollen, AH109 cells transformed with each one of nine candidate substrate‐containing vectors produced significantly higher amounts of β‐galactosidase than cells transformed with the vector not containing *AtSPP* (Fig. 4A). The AT4G32460 signal peptide in cells transformed with the vector not containing *AtSPP* produced some amount of β‐galactosidase, despite the lack of AtSPP expression (Fig. 4A, the gray bar of AT4G32460). It was assumed that because the transmembrane region of the AT4G32460 signal peptide could not anchor to the ER membrane, the N‐terminal tag fused AT4G32460 was released and migrated to the nucleus, where it bound to the Gal4‐responsive promoter. For candidate substrates expressed in the roots, AH109 cells transformed with each one of six candidate substrate‐containing vectors produced significantly higher amounts of β‐galactosidase than cells transformed with the vector not containing *AtSPP* (Fig. 4B). Asterisks in Table 1 indicate signal peptides cleaved by AtSPP. The presence of positively charged amino acids, such as His and Lys, on the C‐terminal side of the signal sequence was noted in the amino acid sequences escaping AtSPP cleavage (Table 1, underbars).

### Repression of AtSPP activity by helix breakers in the substrate

It is known that the flexibility of the transmembrane protein in the lipid bilayer membrane is reduced in conditions where the C‐terminal amino acid exists in a transmembrane domain, because the amino acid strongly interacts with the phospholipids of the membrane [21]. The perpendicular flexibility of the signal peptide influences enzymatic activities. For example, helix breaker in the transmembrane region enhances the cleavage of transmembrane proteins. It is known that Pro affects backbone flexibility because Pro requires bending of the helix axis if it occurs in the interior of a helix [22]. In contrast, side chains of Thr and Ser are back bonded to carbonyl oxygens of the main chain and may induce the structural distortion in the helix backbone [23]. Those distortions can be amplified through the helix, connecting a significant difference of cleavability [24].

**Fig. 5:**
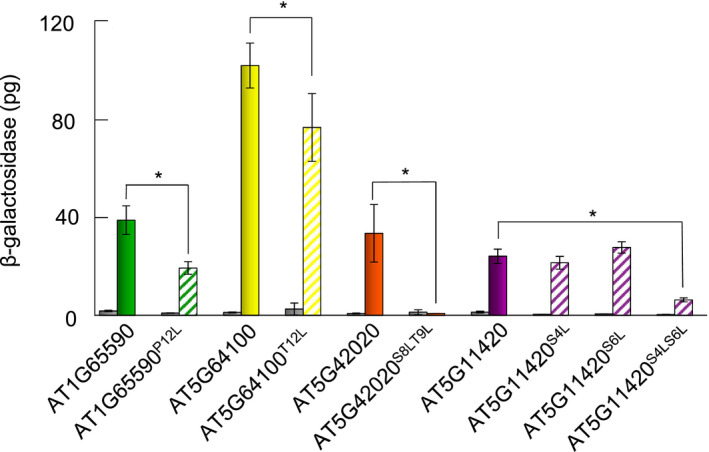
Processing of artificially mutated candidate substrates by AtSPP. Candidate substrates and their mutants were expressed with AtSPP in the yeast‐based reporter assay, and cleavage was quantified as the amount of β‐galactosidase. Gray bars indicate the amount of β‐galactosidase when the p414GDP vector not containing *AtSPP* was used as a control. Green, yellow, orange and purple bars indicate the amount of β‐galactosidase when each candidate substrate or the mutant(s) was processed by AtSPP from the p414GDP vector containing *AtSPP*. Striped bars indicate mutant candidate substrates. The results are presented as the mean ± SEM of four or five trials. The results were analyzed by the two‐sided Welch’s *t*‐test. Asterisks indicate difference with *P < 0.05.

The four candidate substrates, AT1G65590, AT5G64100, AT5G42020 and AT5G11420, have Pro, Thr and Ser in each h‐region and also showed relatively high β‐galactosidase activity (Fig. 4). To confirm that those amino acid residues work as helix breakers, we substituted them to Leu, which would stabilize the helix structure. As shown in Fig. 5, the amount of β‐galactosidase was decreased in cells expressing the Leu mutants of AT1G65590, AT5G64100 and AT5G42020 (Fig. 5, striped bars). There was no decrease in β‐galactosidase observed in cells expressing the single amino acid mutation in AT5G11420, whereas the double substitution of the Ser residues to Leu decreased the amount of β‐galactosidase produced in cells. Thus, the number of helix breaker residues would influence the amount of β‐galactosidase production. These results indicated that the Pro, Thr and Ser amino acids in the AT1G65590, AT5G64100, AT5G42020 and AT5G11420 signal peptides function as helix breakers, and helix breakers in the substrate facilitate the cleavage of AtSPP (Fig. 4 and Table 1, brackets’).

## Discussion

In this study, we successfully constructed a yeast‐based reporter assay for the detection of AtSPP activity. Using this system, we detected proteolytic activity by measuring the amount of β‐galactosidase, whose mRNA was expressed by a peptide fragment after being cleaved by AtSPP and transported to the nucleus. One of the conditions for peptides that were not cleaved by AtSPP could be the presence of the positively charged amino acids His and Lys on the C termini of the signal sequence (Table 1, underbars, and Fig. 4), with the exception of AT3G05490, which was not cleaved by AtSPP despite not having His and Lys in the signal sequence. These results correspond with that of Lemberg [19], who suggested that the existence of Arg downstream of the hydrophobic domain inhibited HsSPP cleavage. The signal peptide AT1G54000 was cleaved by AtSPP, despite having the His residue at the eighth position from the C termini. This suggests that positively charged amino acids located relatively far from the C‐terminus in the peptide could not affect the cleaving activity of AtSPP.

Inhibition of the formation of the enzyme–substrate complex can occur by low flexibility of the peptide in the membrane because of the existence of a positively charged amino acid residue downstream of the hydrophobic helix region, and in this situation, AtSPP would not be able to cleave the peptide.

AtSPP also required the broken‐helix motif in substrates to cleave them, because substitution of the helix breaker to Leu in substrates prevented cleavage by AtSPP (Fig. 5). This trait is similar to that of HsSPP [19, 25, 26]. MHC class I molecules and preprolactin, which are substrates of HsSPP, contain a helix breaker, Ser, in the h‐regions [19, 27], and substitution of Ser to Leu in preprolactin reduces the processing by HsSPP to ~30% of the wild‐type [19]. Thus, binding of the substrate to the active sites of AtSPP is facilitated by the broken‐helix motif in the lipid bilayer. In this study, 15 peptides were cleaved by AtSPP. Amino acid residues related to helix backbone flexibility, such as Ser, Thr, and Pro, would appear statistically one‐fifth of the time in amino acid residues, which indicates the potential of a low substrate specificity of AtSPP.

SPPs are highly expressed in the shoot apical meristem in the seed after 48 h of imbibition in *A. thaliana* and rice, as well as in the embryonic cells, including the roots and the shoot apex [7, 8]. Transmembrane proteins would easily accumulate in those meristematic tissues because those tissues promote cell metabolism and the cell cycle. Therefore, the protease activity of AtSPP with low substrate specificity may positively contribute to membrane protein turnover.

Recent studies suggest that SPP is not only involved in intramembrane proteolysis of signal peptides, but also plays a role in ER‐associated degradation (ERAD) [28, 29]. For example, Chen *et al*. [28] showed that human SPP forms a complex with the ERAD factor Derlin1 and the E3 ubiquitin ligase TRC8 to cleave the unfolded protein response regulator XBP1u. Another example is *Oryza sativa* Derlin 1, which interacts with OsSPP1 and OsSPP2, and acts in the ERAD pathways for ER protein quality control [30]. These findings indicate that AtSPP is also correlated with the ER stress response. Because the AtSPP knockout line in *A. thaliana* is lethal [14], the role of SPP must be pivotal. In contrast, in the *Arabidopsis* database, there are five AtSPP homologs (AtSPPLs). Three of these (AtSPPL1, AtSPPL2 and AtSPPL3) are expressed in the planta. Some AtSPPLs are localized in the endosome [7]. Therefore, the target protein may be different between AtSPP and AtSPPLs. Our reporter assay system of yeast will become a useful tool to determine the innate substrates of plant SPPs and contribute to the identification of the crucial roles of SPP in plants.

## Author contributions

TA, MH, KK and TM conceived and designed research. KK and MH performed experiments. TT, KK and MH analyzed all of the data. TA, TT, KK, MH and KA wrote the manuscript. All authors read and approved the manuscript.

## Conflict of interest

The authors declare no conflict of interest.

## Supporting information

Fig. S1. Screening for genes encoding candidate substrates of AtSPP in the pollen.
Fig. S2. Screening for genes encoding candidate substrates of AtSPP in the roots.
Fig. S3. Detection of AtSPP in overexpressed lines by PCR.
Fig. S4. Detection of AtSPP in knockdown lines by PCR.
Table S1. Primer sequences for PCR to amplify the target gene fragments.
Table S2. Primer sequences for PCR to amplify the gene fragments encoding candidate substrates of AtSPP.
Table S3. Primer sequences for PCR to produce artificially mutated substrates.

## Data Availability

The datasets generated and/or analyzed during the current study are available in the Gene Expression Omnibus (GEO) database (https://www.ncbi.nlm.nih.gov/geo/) under accession number GSE129113. All data generated or analyzed during this study are included in this published article (and its Supplementary Information files). The raw data are available from the corresponding author upon reasonable request.
