# Supplementary material for: Yeast‐based reporter assay system for identifying the requirements of intramembrane proteolysis by signal peptide peptidase of Arabidopsis thaliana
Source: FEBS Open Bio. 2020 Aug 7;10(9):1833–42. doi: 10.1002/2211-5463.12936 (PMC7459403; doi:10.1002/2211-5463.12936)
Supplement: Supplementary file 1 — Fig. S1. Screening for genes encoding candidate substrates of AtSPP in the pollen. Fig. S2. Screening for genes encoding candidate substrates of AtSPP in the roots. Fig. S3. Detection of AtSPP in overexpressed lines by PCR. Fig. S4. Detection of AtSPP in knockdown lines by PCR. Table S1. Primer sequences for PCR to amplify the target gene fragments. Table S2. Primer sequences for PCR to amplify the gene fragments encoding candidate substrates of AtSPP. Table S3. Primer sequences for PCR to produce artificially mutated substrates. [file FEB4-10-1833-s001.pdf]

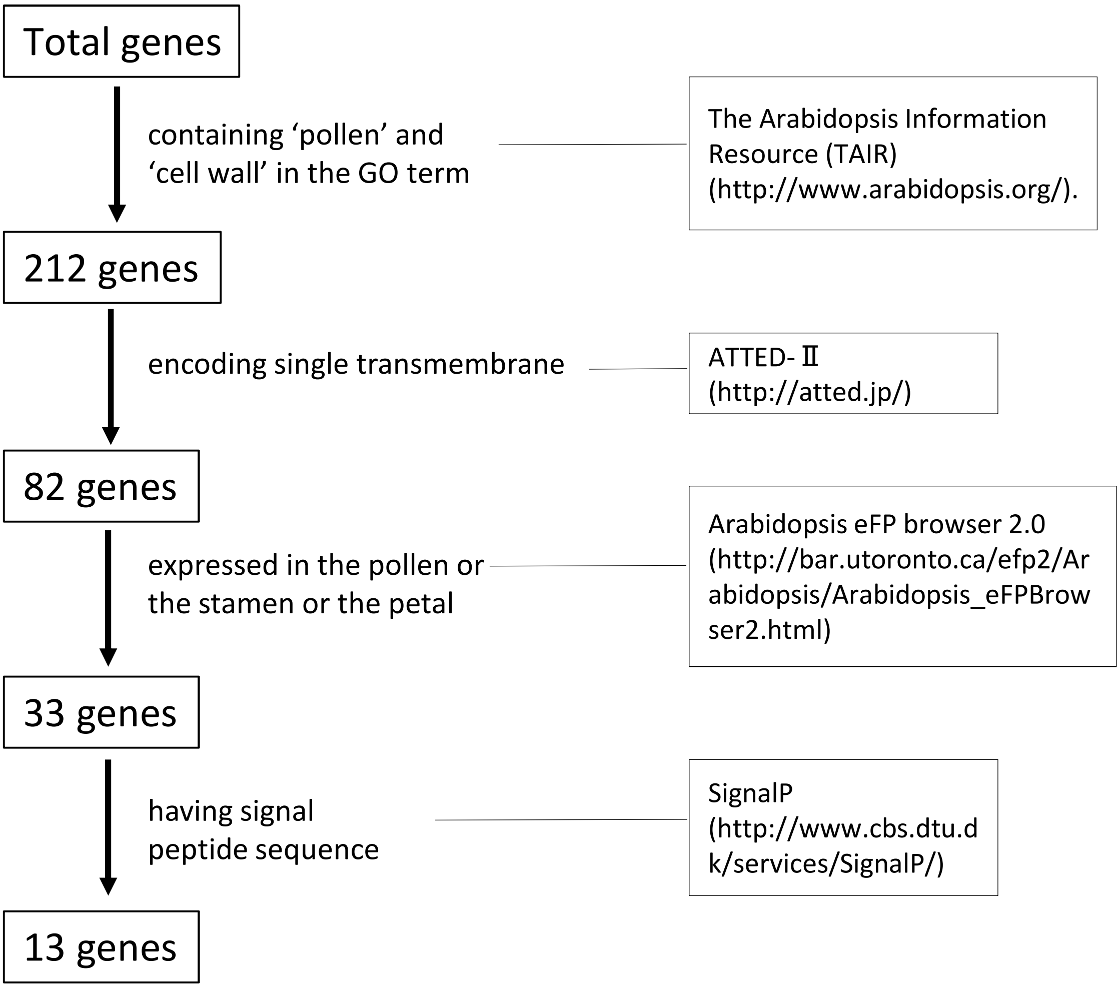

Figure S1 Screening for genes encoding candidate substrates of AtsPP in the pollen

In accordance with the flow chart, we first selected 212 genes containing 'pollen' and 'cell wall' in the GO term from the TAIR database (The Arabidopsis Information Resource, http://www.arabidopsis.org/). Next, the 82 genes which encode single transmembrane proteins were chosen using the ATTED-Ⅱ database (http://atted.jp/). Then the 33 genes that were expressed in the pollen, the stamen or the petal were picked using the Arabidopsis eFP browser 2.0 (http://bar.utoronto.ca/efp2/Arabidopsis/Arabidopsis_eFPBrowser2.html). Finally, using the SignalP database (http://www.cbs.dtu.dk/services/SignalP/), 13 genes whose proteins had signal peptide sequence were targeted as the candidate substrates of AtSPP in the pollen.

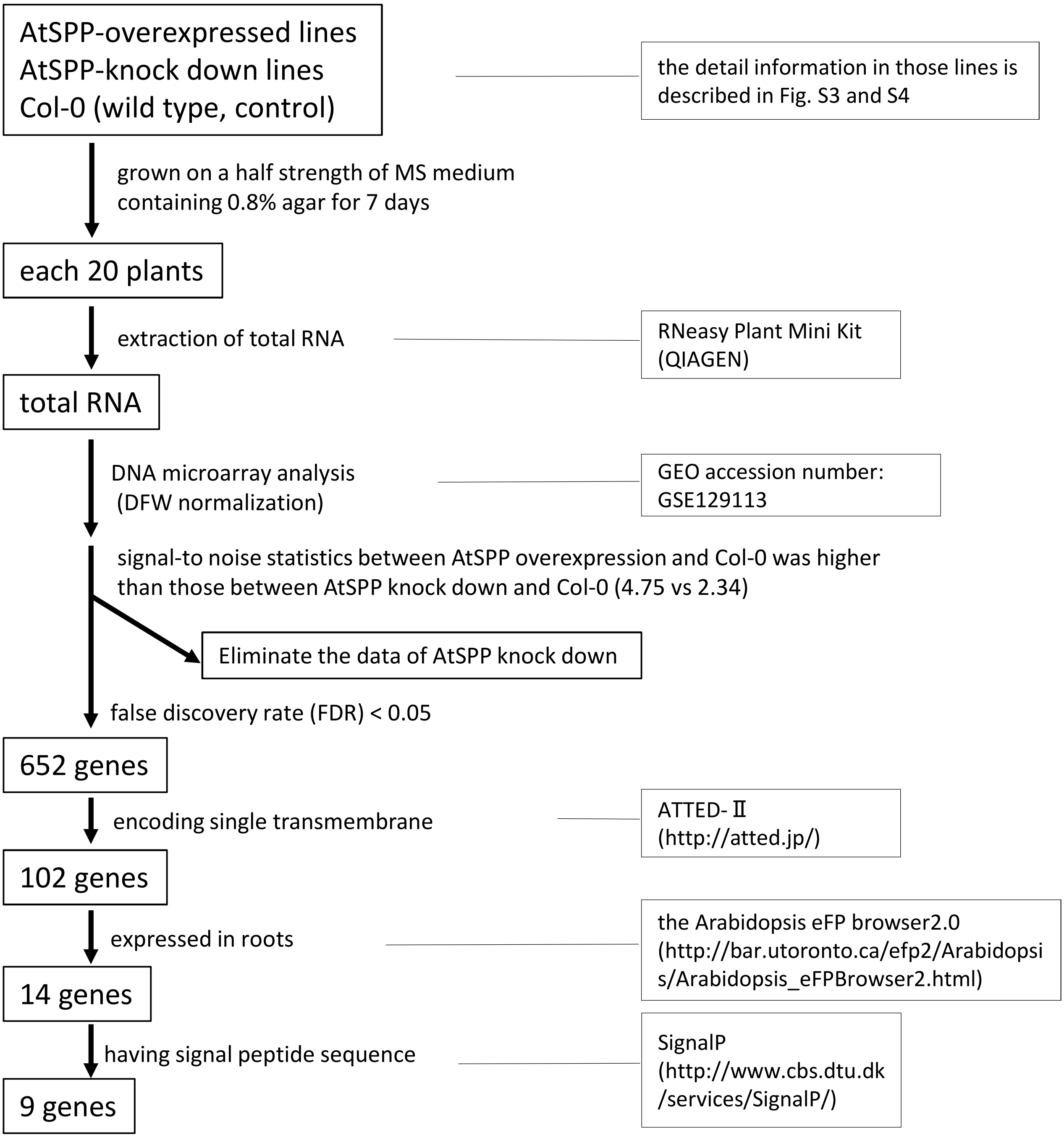

Figure S2 Screening for genes encoding candidate substrates of AtSPP in the roots

In accordance with the flow chart, we first created the AtSPP-overexpressed lines and the knock down lines (Fig. S3 and S4). For the transcriptome analysis, the Col-0 strain and the AtSPP-overexpressed lines were grown on a half strength of MS medium containing 0.8% agar for 7 days, and the total RNA was extracted by RNeasy Plant Mini Kit (QIAGEN). A DNA microarray was performed using Affymetrix ATH1 arrays (GSE129133). After normalization of the signals with the DFW algorithm using the statistical language R, the expression levels in the Col-0 strain and the AtSPP-overexpressed lines were compared by the "Rank products" function. The 652 genes whose false discovery rate (FDR) values were less than 0.05 in gene expression level were extracted. Next, the 102 genes which encode single transmembrane proteins were extracted by the ATTED-Ⅱ database. Then, the 14 genes expressed in the roots were selected with the Arabidopsis eFP browser2.0 (http://bar.utoronto.ca/efp2/Arabidopsis/Arabidopsis_eFPBrowser2.html). Finally, the 9 genes encoding proteins having signal peptide sequence were selected using SignalP database (http://www.cbs.dtu.dk/services/SignalP/). The signal sequences encoded by the 9 genes were defined as the candidate substrates of AtSPP in the roots.

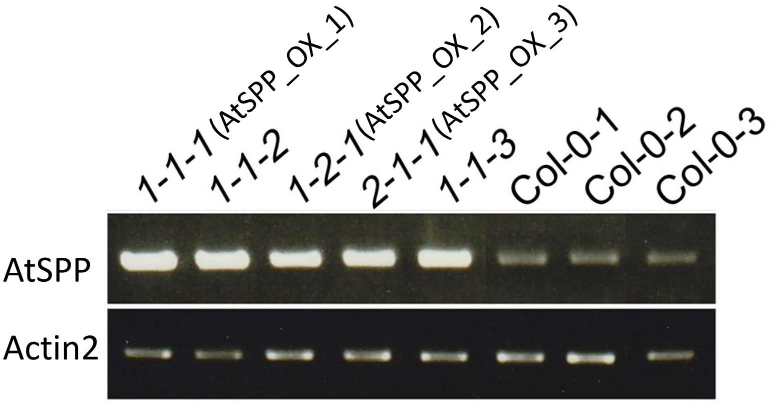

1% agarose gel,
ethidium bromide staining

Figure S3 Detection of AtSPP in overexpressed lines by PCR

The full length *AtSPP* was cloned under CaMV 35S and Nos-T with *Xba* I and *Sac* I in a pZH2B binary vector. Then the vector was introduced to the agrobacterium, and the agrobacterium infected wild-type *A. thaliana* plants (Col-0). The homozygous transgenic plants were regarded as AtSPP-overexpressed lines.

The photos showed *AtSPP* fragments in cDNAs from the AtSPP-overexpressed lines, amplified by PCR with 23 cycles (annealing at 62℃, elongation at 72℃, denaturation at 94 ℃, each for 30 sec). Primer sequences for this PCR were as follows; 5’-TTATTTCAAGTCTTTGGAGGTAGAG-3’ and 5’- ACCGTTCCAAATGCAGTGAGAAG-3’ were used for *AtSPP* amplification, and 5’- ACCTTGCTGGACGTGACCTTACTGAT-3’, and 5’-GTTGTCTCGTGGATTCCAGCAGCTT-3’ were used for *Actin 2* amplification.

A

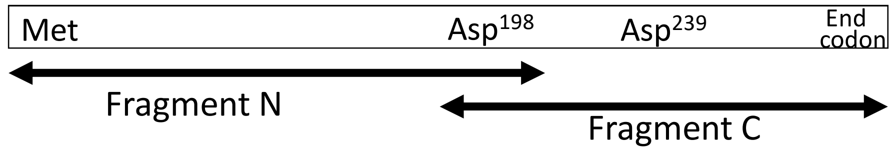

Fragment N: the 600 bp fragment containing Asp[198] in *AtSPP*

Fragment C: the 400 bp fragment containing Asp[198] and Asp[239] in *AtSPP*

B

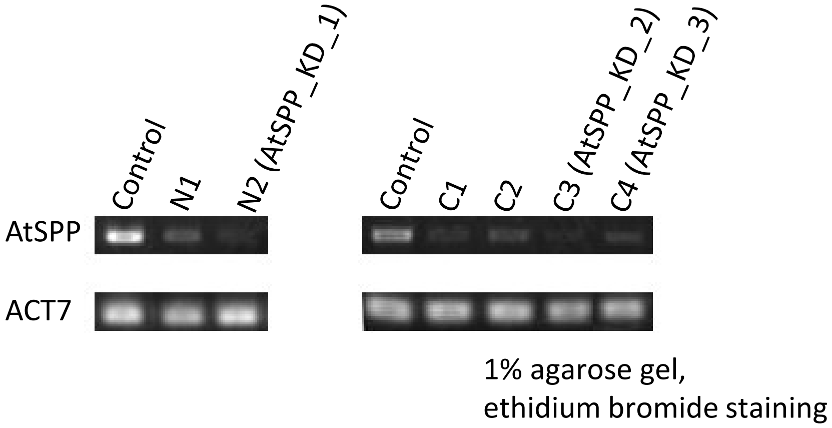

Figure S4 Detection of AtSPP in knock down lines by PCR

A: The fragments N and C were cloned into the pBI-sense, anti sense-GW vector. Each vector was introduced to agrobacterium, and the agrobacterium infected wild-type *A. thaliana* plants (Col-0). The homozygous transgenic plants were regarded as AtSPP-RNAi lines.

B: *AtSPP* fragments in cDNAs from the AtSPP-RNAi lines were amplified by PCR with 23 cycles (annealing at 62 ℃, elongation at 72 ℃, denaturation at 94 ℃, each for 30 sec). Primer sequences for this PCR were as follows; 5′-TTATTTCAAGTCTTTGGAGGTAGAG-3′ and 5′- ACCGTTCCAAATGCAGTGAGAAG-3′ were used for *AtSPP* amplification, and 5′-TGCTGACCGTATGAGCAAAG-3′, and 5′-ATCCTCCGATCCAGACACTG-3′ were used for *ACT7* amplification.

Table S1. Primer sequences for PCR to amplify the target gene fragments

Target	Primer sequence
AtSPP	5'-AggatccATGAAGAATTGTGAG-3'
	5'-CgaattcTCATTCATCATGAGC-3'
HsSPP	5'-AggatccATGGACTCGGCCC-3'
	5'-CgaattcTCATTTCTCTTTCTTC-3'
AtSPP^{D198A}	5’-AGCATAGAAAAATAGTCCTGCCAAAAGG-3’
	5’-ATCTTCTGGGTTTTCTTTACTCCAGTTATG -3’
AtSPP^{D198A,D239A}	5’-GGCACCAAGACCAAGCATAGAATAGG-3’
	5’-ATTGTCATTCCGGGTATTTTCGTTG-3’
gpUL40^{T31IT33}	5’-AGAATTAGATCTTTGTTGTGAAGC-3'
	5’-CATACACAACAAACCAACAGTC-3'
preprolactin	5’-CggatccATGGACAGCAAAGGTTCG-3'
	5’-CaagcttGGAGGGCGGACCCTG-3'

The upper primer was the sense strand primer and the lower primer was the antisense strand primer in each target. Restriction enzyme sequences are shown in lowercase letters.

Table S2. Primer sequences for PCR to amplify the gene fragments encoding candidate substrates of AtSPP

Localization: pollen, stamen, or petal	
AGI code	Primer sequence
AT2G06850	5'-CAggatccACTGTTTCTTCATCTCCAT-3'
	5'-CAaagcttAGGAATAGCCATTACCATTGTT-3'
AT5G49360	5'-CCggatccTCTTGTTATAATAAAGCACTA-3'
	5'-CAaagcttTGACTCTGACGAGTGAAC-3'
AT1G65590	5'-CAggatccAGAGGTAGCGGAGCAAAG-3'
	5'-CAaagcttGGCGGAGATTGTTCCG-3'
AT5G07410	5'-CCggatccAGATACACAAATGTGTCCATT-3'
	5'-CAaagcttTGCGAACACCATTGGTGAAAC-3'
AT1G69940	5'-CAggatccGGATACACAAATGTGTCCATT-3'
	5'-CAaagcttTGCGAACACCATCGGTGTA-3'
AT5G54570	5'-CAggatccGAATCTTTAATGAGACTCGTC-3'
	5'-CAaagcttTGAAGAAACGTGATCAAGAGG-3'
AT3G26720	5'-CAggatccGCGGTCAAATGTTTCTCTCT-3'
	5'-CAaagcttGGAGGTGACGCCGCCGATTA-3'
AT5G25460	5'-CAggatccGAAGGCGTCACCGTCGT-3'
	5'-CAaagcttGGCCATGGCGGTGGCG-3'
AT5G11420	5'-CAggacttAAAGGAGGCAGCCTCTCGTT-3'
	5'-CAaagcttGCAAATGACGGAAGTGATGGT-3'
AT4G25900	5'-CAggatccATGGGGAATAAGCGAAATCTC-3'
	5'-CAaagcttGGCTACTACTGCAACCACCAG-3'
AT5G21100	5'-CAggatccGCGGTAATTGTGTGGTGGCTA-3'
	5'-CAaagcttCGCCGACGCCGAGTGAAAC-3'
AT5G12950	5'-CAggatccAAGTCTGGTCTCATCATAACC-3'
	5'-CAaagcttCGCTACAGAAACAAGAACAAA-3'
AT4G32460	5'-CAggatccAAAGAGATGGGAGTGATAGTG-3'
	5'-CAaagcttGAAGCAAAAGGCAACGTAGAA-3'
Localization: roots	
AGI code	Primer sequence
AT4G33720	5'-CCggatccAAAATCTTTAACTCATCTCAA-3'
	5'-CCaagcttGGCTTTTAGATGAACTATGAG-3'
AT2G46330	5'-CAggatccGCGTCGAGAAACTCCGTCA-3'
	5'-CAaagcttAGCGCCGGCGAGAGATAAGA-3'
AT4G25810	5'-CAggatccGCGATGATCAGTTACTCCACC-3'
	5'-CCaagcttGGCGGAAACAGAGCAAATCAT-3'
AT5G61790	5'-CAggatccAGACAACGGCAACTATTTTCC-3'
	5'- CCaagcttACAGTAGCAAAGCTTCTGGAA-3'
AT5G64100	5'-CAggatccGGTCGTGGTTACAATTTGCTA-3'
	5'-CCaagcttTGCGGTTACAGCTGCAACCAA-3'
AT5G42020	5'-CAggatccGCTCGCTCGTTTGGAGCAA-3'
	5'-CCaagcttTGCAGTGGAAAACGCAAATA-3'
AT3G05490	5'-CAggatccACGAACACTCGCGCGATCTAC-3'
	5'-CCaagcttTGATTCCACGGCTGAGATTAC-3'
AT4G12510	5'-CAggatccGCTTCAAAAATTTCAGCCTCT-3'
	5'-CCaagcttTGCGGTGGTTAAAGTGAAAAA -3'
AT1G54000	5'-CCggatccATGGCAAACAACTGTAATTTA-3'
	5'-CAaagcttTCCGGCGACAGTGATGGG-3'

The upper primer was the sense strand primer and the lower primer was the antisense strand primer in each target.
Restriction enzyme sequences are shown in lowercase letters.

Table S3. Primer sequences for PCR to produce artificially mutated substrates

AGI code with mutaion	Primer sequence
AT1G65590^{P12L}	5'-TTGTTTATGCTATTCATCGCCGGAACAATC-3'
	5'-CAACAAAACGCCGGCGATCTTTGCTCCGC-3'
AT5G64100^{T12L}	5'-TTGTTTTTAGTATTGGTTGCAGCTGTAACC-3'
	5'-TACTAGAACGAATAGCAAATTGTAACCACGAC-3'
AT5G42020^{S8LT9L}	5'-GTTGTGCTTGCGATCATCTTCTT-3'
	5'-CAACAAGTTTGCTCCAAACGAGC-3'
AT5G11420^{P4L}	5'-TTTCTCTTCGTTCTCCTAATCGCCACCAT-3'
	5'-CGAGAGCAAGCCTCCTTTGGATCCCTCTT-3'
AT5G11420^{P6L}	5'-TTTCTCTTCGTTCTCCTAATCGCCACCAT-3'
	5'-CAAGAGGCTGCCTCCTTTGGATCCCTCTT-3'
AT5G11420^{P4LP6L}	5'-TCTTGTTTCTCTTCGTTCTCCTAATCGCCA-3'
	5'-GCAAGCCTCCTTTGGATCCCTCTTTTTTTG-3'

The upper primer was the sense strand primer and the lower primer was the antisense strand primer in each target.
Substituted bases for point mutation are indicated by red letters.
